# Genomic Characterization of Extensively Drug-Resistant NDM-Producing *Acinetobacter baumannii* Clinical Isolates With the Emergence of Novel *bla*_ADC-257_

**DOI:** 10.3389/fmicb.2021.736982

**Published:** 2021-11-22

**Authors:** Mai M. Zafer, Amira F. A. Hussein, Mohamed H. Al-Agamy, Hesham H. Radwan, Samira M. Hamed

**Affiliations:** ^1^Department of Microbiology and Immunology, Faculty of Pharmacy, Ahram Canadian University, Cairo, Egypt; ^2^Clinical and Chemical Pathology Department, Faculty of Medicine, Cairo University, Cairo, Egypt; ^3^Department of Pharmaceutics, College of Pharmacy, King Saud University, Riyadh, Saudi Arabia; ^4^Department of Microbiology and Immunology, Faculty of Pharmacy, Al-Azhar University, Cairo, Egypt; ^5^Department of Microbiology and Immunology, Faculty of Pharmacy, October University for Modern Sciences and Arts (MSA), Giza, Egypt

**Keywords:** healthcare-associated infections, *Acinetobacter baumannii*, extensive drug resistance, *bla*_NDM_, whole-genome sequencing, multilocus sequence typing

## Abstract

*Acinetobacter baumannii* has become a major challenge to clinicians worldwide due to its high epidemic potential and acquisition of antimicrobial resistance. This work aimed at investigating antimicrobial resistance determinants and their context in four extensively drug-resistant (XDR) NDM-producing *A. baumannii* clinical isolates collected between July and October 2020 from Kasr Al-Ainy Hospital, Cairo, Egypt. A total of 20 *A. baumannii* were collected and screened for acquired carbapenemases (*bla*_NDM_, *bla*_VIM_ and *bla*_IMP_) using PCR. Four NDM producer *A. baumannii* isolates were identified and selected for whole-genome sequencing, *in silico* multilocus sequence typing, and resistome analysis. Antimicrobial susceptibility profiles were determined using disk diffusion and broth microdilution tests. All *bla*_NDM_-positive *A. baumannii* isolates were XDR. Three isolates belonged to high-risk international clones (IC), namely, IC2 corresponding to ST570^Pas^/1701^Oxf^ (M20) and IC9 corresponding to ST85^Pas^/ST1089^Oxf^ (M02 and M11). For the first time, we report *bla*_NDM-1_ gene on the chromosome of an *A. baumannii* strain that belongs to sequence type ST164^Pas^/ST1418^Oxf^. Together with *AphA6*, *bla*_NDM-1_ was bracketed by two copies of IS*Aba14* in ST85^Pas^ isolates possibly facilitating co-transfer of amikacin and carbapenem resistance. A novel *bla*_ADC_ allele (*bla*_ADC-257_) with an upstream IS*Aba1* element was identified in M19 (ST/CC164^Pas^ and ST1418^Oxf^/CC234^Oxf^). *bla*_ADC_ genes harbored by M02 and M11 were uniquely interrupted by IS1008. Tn*2006*-associated *bla*_OXA-23_ was carried by M20. *bla*_OXA-94_ genes were preceded by IS*Aba1* element in M02 and M11. AbGRI3 was carried by M20 hosting the resistance genes *aph*(*3`*)*-Ia*, *aac*(*6`*)*-Ib`*, *catB8*, ant(3*``*)*-Ia*, *sul1*, *armA*, *msr*(*E*), and *mph*(*E*). Nonsynonymous mutations were identified in the quinolone resistance determining regions (*gyrA* and *parC*) of all isolates. Resistance to colistin in M19 was accompanied by missense mutations in *lpxACD* and *pmrABC* genes. The current study provided an insight into the genomic background of XDR phenotype in *A. baumannii* recovered from patients in Egypt. WGS revealed strong association between resistance genes and diverse mobile genetic elements with novel insertion sites and genetic organizations.

## Introduction

Hospital-associated infections (HAIs) present an elevated healthcare burden in both developed and developing countries (Chng et al., 2020). *Acinetobacter baumannii* is implicated in a considerable fraction of difficult to treat HAIs (Ayobami et al., 2019). Antimicrobial resistance, biofilm formation, and resistance to desiccation are among the competencies contributing to the environmental persistence and the epidemic potential of this species (Antunes et al., 2014). In addition to its intrinsic resistance to multiple antimicrobial classes, effective therapeutic options are being gradually depleted by the extraordinary ability of *A. baumannii* to acquire and upregulate resistance genes (Di Nocera et al., 2011). The emergence of multidrug-resistant (MDR) and extensively drug-resistant (XDR) *A. baumannii* has been increasing worldwide as well as in Egypt (Tal-Jasper et al., 2016; Elsayed et al., 2020). This forced the WHO to declare carbapenem-resistant *A. baumannii* as a category 1 (critical) priority pathogen for which novel therapeutic antimicrobials are urgently required (Tacconelli et al., 2018).

The New Delhi Metallo-β-lactamase-1 (NDM-1) is a carbapenemase that has been frequently linked to the XDR phenotype owing to its association with mobile elements loaded with other resistance genes (Wailan and Paterson, 2014). *A. baumannii* has been long recognized as an intermediate reservoir for *bla*_NDM-1_ genes in which the harboring transposon (Tn*125*) was built and subsequently transmitted to other Gram-negative species (Toleman et al., 2012; Bontron et al., 2016).

Genome studies contribute significantly to better comprehend the molecular basis and evolution dynamics of antimicrobial resistance in nosocomial infectious pathogens (Hendriksen et al., 2019). Despite the large number of studies from Egypt that have discussed the epidemiology of healthcare-associated *A. baumannii* (Al-Hassan et al., 2019; Benmahmod et al., 2019; Wasfi et al., 2021), few studies have explored the whole-genome sequence of those circulating in Egyptian hospitals (Fam et al., 2020).

The objective of the current study was to explore the genomic features of four XDR *bla*_NDM_*-*positive *A. baumannii* clinical isolates recovered from hospitalized patients at a large tertiary hospital in Egypt by whole-genome sequencing (WGS).

## Materials and Methods

### Bacterial Strains and Antimicrobial Susceptibility Testing

A total of 54 nonduplicate nonfermentative Gram-negative bacterial isolates were collected from Kasr Al-Ainy University Hospital, Cairo, Egypt, between July and October 2020. Of these, 20 isolates were identified as *A. baumannii* using VITEK 2 (bioMérieux, Marcy l’Etoile, France). The identity of *A. baumannii* isolates was further confirmed using PCR amplification of the *bla_OXA-51-like_* genes (Turton et al., 2006). Bacterial isolates were recovered at the clinical pathology laboratory as part of routine clinical care of hospitalized patients. Antimicrobial resistance profiles were identified using disk diffusion test according to the recommendations of the CLSI (2018). Tigecycline susceptibility test results were interpreted according to susceptibility breakpoints recommended by the EUCAST (2021) v11.0 for *Enterobacterales*. For disk diffusion test, 14 antimicrobial disks (Oxoid, United Kingdom) were used including the following: ampicillin (10μg), amoxicillin/clavulanic acid (20/10μg), piperacillin/tazobactam (10/100μg), ceftriaxone (30μg), cefoxitin (30μg), cefepime (30μg), cefotaxime (30μg), levofloxacin (5μg), imipenem (10μg), meropenem (10μg), amikacin (30μg), tigecycline (15μg), and trimethoprim/sulfamethoxazole (1.25/23.75μg). The broth microdilution method was used to detect the minimum inhibitory concentration (MIC) of colistin according to CLSI guidelines. Amplification of MBL genes (*bla*_NDM_, *bla*_VIM,_ and *bla*_IMP_) using polymerase chain reaction (PCR) was done for all *A. baumannii* isolates as previously described (Ghazawi et al., 2012). Individual *A. baumannii* isolates (M02, M11, M19, and M20) that harbored *bla*_NDM_ were selected for WGS analysis.

### Whole-Genome Sequencing, Assembly, and Annotation

DNA was extracted from all *bla*_NDM_-positive *A. baumannii* isolates using QIAGEN DNA purification kit (Qiagen, Valencia, CA). This was further manipulated by Nextera DNA Sample Preparation kit (Nextera, United States) for preparation of the DNA library according to the manufacturer’s recommended protocol. Sequencing was performed using the paired end 2×150bp reads sequencing technology on an Illumina MiSeq platform (Illumina Inc., San Diego, CA, United States). Reads quality was assessed using FastQC v0.11.9 (Brown et al., 2017) before trimming with Trimmomatic v0.35 to cut away remaining adaptors and low-quality reads (Bolger et al., 2014). Trimmed reads were *de novo* assembled using SPAdes 3.14.1 (Bankevich et al., 2012) with default parameters. The quality of genomes assembly was evaluated using QUAST v5.0.2 (Gurevich et al., 2013). Functional annotations of the draft genomes were generated during submission to the National Center for Biotechnology Information (NCBI) genome database using the NCBI Prokaryotic Genome Annotation Pipeline (PGAP; Tatusova et al., 2016). Plasmid sequences were identified using plasmidSPAdes (Antipov et al., 2016) and Unicycler (Wick et al., 2017) for raw reads assembly and Bandage (Wick et al., 2015) for visualization of circular contigs.

### Multilocus Sequence Typing

Whole-genome sequencing data were used for *in silico* analysis of multilocus sequence types (MLSTs) of the isolates harboring *bla*_NDM_ gene based on both Pasteur and Oxford schemes. Allele numbers and sequence types (STs) were assigned using PubMLST server.^1^ The global optimal eBURST (goeBURST) algorithm executed by PHYLOViZ V2.0 (Francisco et al., 2012) was used for constructing a complete minimum spanning tree (MST) of the sequence types of the *bla*_NDM_-positive isolates together with other STs in MLST database (accessed on March 10, 2021), and clonal complexes (CCs) were assigned accordingly.

### Phylogeny Analysis

A single nucleotide polymorphism (SNP)-based phylogeny analysis of the four *bla_NDM_*-positive isolates was performed using the CSI-Phylogeny tool hosted by the CGE server (Center for Genomic Epidemiology, Lyngby, Denmark) available at http://www.genomicepidemiology.org/ (Kaas et al., 2014). The draft genomes of the isolates were compared to complete genomes of *A. baumannii* strains carrying *bla*_NDM-1_ gene and some *A. baumannii* strains that belong to high-risk international clones retrieved from the GenBank database (accessed in: October 12, 2021). In addition, draft genomes of *A. baumannii* strains that belong to ST1418^Oxf^ and ST164^Pas^ were also downloaded from PubMLST genome collection^2^ and included in the analysis. A*. baumannii* ATCC 17978 was used as a reference genome. The phylogenetic tree was visualized and edited using the interactive tree of life v3 software (Letunic and Bork, 2016) available at: https://itol.embl.de/.

### Analysis of Antimicrobial Resistance Determinants and Insertion Sequences

Acquired antimicrobial resistance genes were identified using the ResFinder 4.1 webtool on the CGE server (Center for Genomic Epidemiology, Lyngby, Denmark^3^; Bortolaia et al., 2020) using raw reads as an input. Assembled contigs were further analyzed using the Comprehensive Antibiotic Resistance Database server^4^ (Alcock et al., 2020) with coverage and identity thresholds of 80 and 95%, respectively. Genomic resistance islands were predicted using IslandViewer4 webtool^5^ (Bertelli et al., 2017). Gene mutations relevant to antimicrobial resistance were manually analyzed by extracting the genes of interest from genome assemblies and blasting against respective genes of the reference strain *A. baumannii* ATCC 19606 (Accession Number: CP045110.1). This involved analysis of *gyrA* and *parC* regions whose mutations are associated with quinolones resistance. In addition, other genes reported to affect the susceptibility of *A. baumannii* to colistin including those involved in lipid A biosynthesis pathway (*lpxA*, *lpxC*, and *lpxD*) and *pmrABC* operon were also analyzed in case of colistin nonsusceptibility. Insertion sequences (ISs) were identified using the online tool ISfinder^6^ (Siguier et al., 2006).

### Characterization of the Genetic Context of Resistance Genes

Contigs containing resistance genes were extracted from the assemblies. Genetic features were obtained from PGAP annotation data. Unannotated regions were manually reannotated after blasting against the GenBank nucleotide collection. Genetic environments of resistance gene cassettes located on more than one contig were identified by mapping of raw reads to the best hits of the contigs’ blast analyses using BWA (Li and Durbin, 2009). Consensus sequences were obtained using SAMtools and bcftools v0.1.10 (Li, 2011). Finally, annotated genetic environments of resistance genes were visualized using SnapGene viewer v5.1.3.1 (from Insightful Science; available at snapgene.com) and compared to reference sequences using Easyfig v2.2.5 (Sullivan et al., 2011).

### Nucleotide Sequence Accession Numbers

Raw reads obtained by WGS of the *bla*_NDM_-positive isolates were submitted to the Sequencing Read Archive^7^ of the NCBI. Draft genomes were submitted to the NCBI Genome database.^8^ Together with their BioSamples, they were submitted under the BioProject number PRJNA690827. Raw reads and draft genomes accession numbers are shown in Supplementary Table 1. The nucleotide sequence of the novel *bla*_ADC–257_ gene was deposited in the NCBI GenBank database under the accession number (MZ224611.1).

## Results

During the study period, a total of 20 *A. baumannii* isolates were recovered from 20 different hospitalized patients with age ranging between newborn (5days) and 65years old. Of these, 12 (60%) were females and 8 (40%) were males. More than half of the patients were hospitalized in intensive care units. Specimens were collected from different clinical sites (Table 1). Results are shown for the four *bla*_NDM_-positive *A. baumannii* isolates.

**Table 1:** Clinical data of the four NDM-producing *Acinetobacter baumannii*.

Isolate	Site	Age	Sex	Diagnosis	Date of isolation	Hospital ward
M02	Wound	28years	Female	Subovarian abscess removal	2020-07-10	ICU
M11	Pleural	20days	Female	Pneumonia	2020-07-15	NICU
M19	Blood	20years	Female	Fever of unknown origin	2020-10-20	ICU
M20	Blood	65years	Male	Splenectomy and feverish	2020-08-2	ICU

ICU, intensive care unit; NICU, neonatal intensive care unit.

### *Acinetobacter baumannii* Strains Harboring *bla*_NDM_ Gene

To determine the prevalence of acquired carbapenemases in the recovered *A. baumannii* isolates, the presence of *bla*_NDM_, *bla*_VIM,_ and *bla*_IMP_ genes were assessed using PCR assay. Neither VIM- nor IMP-type carbapenemase-coding genes could be identified in the isolates. Out of 20 *A. baumannii* isolates, four (20%) showed amplification of 371bp PCR product corresponding to *bla*_NDM_ gene.

### Genome Assembly

Whole-genome sequencing of the *bla*_NDM_-positive isolates yielded total assembly lengths ranging from 3,773,846bp to 3,919,334bp with a GC content ranging from 39.19 to 39.55%. The mean number of contigs was 633. The number of coding sequences predicted by PGAP annotation of the assembled contigs ranged from 3,761 to 3,996. Post-assembly and annotation metrics of the *bla*_NDM_-positive isolates are shown in Supplementary Table 2.

### MLST and Phylogenetic Analysis

**Figure 1:**
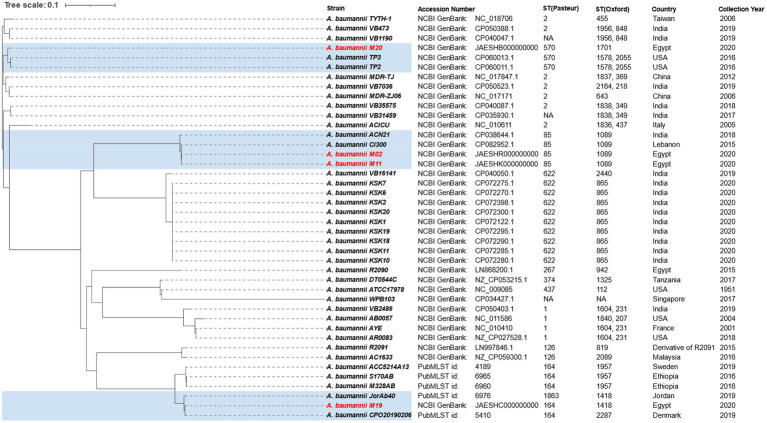
A single nucleotide polymorphism-based phylogenetic tree depicting the genetic relatedness of *bla*_NDM-1_-positive isolates sequenced in the current study to other *Acinetobacter baumannii* strains. *A. baumannii* strains sequenced in the current study together with their genetically related strains are highlighted by blue color.

*In silico* MLST analysis of the *bla*_NDM_-positive isolates and goeBURST analysis of their STs together with ST data from MLST database revealed that isolate M20 (ST570^Pas^/1701^Oxf^) belongs to clonal complex (CC2^Pas^/546^Oxf^) representing international (IC) 2. Two isolates M02 and M11 had the same sequence type (ST85^Pas^/ST1089^Oxf^) that was found to belong to CC464^Pas^/CC1078^Oxf^ classified within IC9. The allele profile of M19 matched ST/CC164 and ST1418/CC234, according to Pasteur and Oxford schemes, respectively. MST diagram of *bla*_NDM_-positive isolates STs together with other STs in MLST database (Pasteur scheme) is shown in Supplementary Figure 1. A SNP-based phylogenetic tree depicting the genetic relatedness of our *bla*_NDM_-positive isolates to other *A. baumannii* strains is shown in Figure 1.

### Antimicrobial Susceptibility Testing and Resistance Determinants

**Figure 2:**
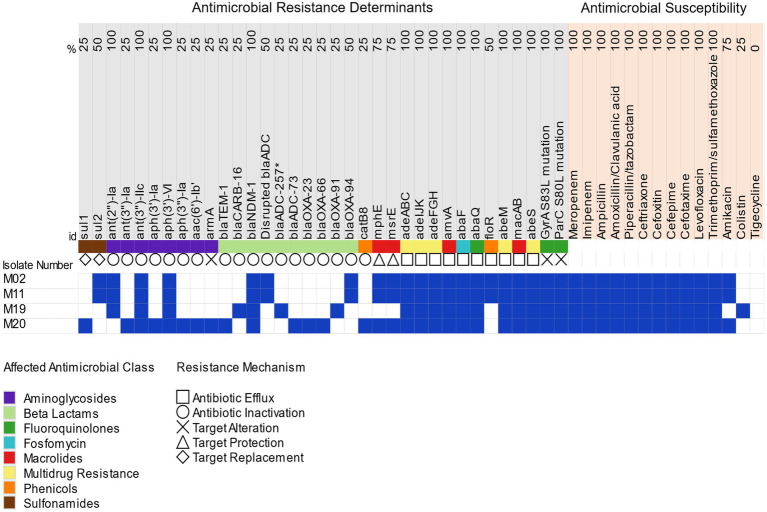
Antimicrobial resistance profiles and distribution of antimicrobial resistance determinants in the *bla*_NDM_-positive isolates. Blue colors denote antimicrobial resistance and harbored antimicrobial determinants, while susceptibility to antimicrobials and lack of resistance determinants are denoted by white colors; *bla*_ADC_-257^*^, novel *bla*_ADC_ allele identified in M19.

Antimicrobial susceptibility testing revealed that all isolates were extensively drug resistant (XDR) with retained susceptibility to only two antimicrobial classes (Magiorakos et al., 2012; Figure 2). All isolates were susceptible to tigecycline. MIC values of ≤0.125, 0.25, ≥128, and 0.5μg/ml were determined for colistin in M02, M11, M19, and M20, respectively. Resistance to colistin was shown by one isolate (M19) that also retained susceptibility to amikacin.

Investigating the genetic background of the XDR phenotype using WGS revealed that the isolates carried multiple acquired antimicrobial resistance determinants besides the intrinsic resistance genes (Table 2). Genes conferring resistance to β-lactams included class A β-lactamases (*bla*_CARB-16_ and *bla*_TEM-1_), one metallo-β-lactamase (*bla*_NDM-1_), class C β-lactamases (*bla*_ADC-73_ and *bla*_ADC-257_), and carbapenem-hydrolyzing Ambler class D β-lactamases, (*bla*_OXA-23_, *bla*_OXA-66_, *bla*_OXA-91_, and *bla*_OXA-94_). *bla*_ADC-257_ is a novel allele of *bla*_ADC-52_ (GenBank accession: WP_001211232.1) detected in isolate M19 with two amino acid substitutions (R2Q and G24D). Resistance to other antimicrobial agents was conferred by *ant*(*2″*)*-Ia*, *ant*(*3″*)*-Ia*, *ant*(*3″*)*-IIc*, *aph*(*3′*)*-Ia*, *aph*(*3″*)*-Ia*, *aph*(*3′*)*-VI*, *aac*(*6′*)*-Ib’*, and *ArmA* (aminoglycoside resistance), *mphE* and *msrE* (macrolide resistance), *catB8* (chloramphenicol resistance), and *sul1* and *sul2* (sulfonamide resistance).

**Table 2:** STs and antimicrobial resistance genes carried by the four *bla*_NDM_-positive isolates.

Isolate number	MLST				Intrinsic *bla*_OXA_ gene	Antimicrobial resistance genes	Efflux pumps genes	QRDR^b^	
	Pasteur		Oxford					*gyrA*	*parC*
	ST	CC	ST	CC					
M02	85	464	1089	1078	*bla*_OXA-94_	*aph*(*3′*)*-VI*, *bla*_NDM-1_, *bla*_ADC_ (disrupted by IS6), *mphE*, *msrE*, *sul2*, *ant*(*2″*)*-Ia*, *ant*(*3″*)*-IIc*	*adeABC*, *adeIJK*, *adeFGH*, *abeM*, *amvA*, *abeS*, *abaF*, *abaQ*, *floR*, *macAB*	S83L	S80L
M11	85	464	1089	1078	*bla*_OXA-94_	*aph*(*3′*)*-VI*, *bla*_NDM-1_, *bla*_ADC_ (disrupted by IS6), *mphE*, *msrE*, *sul2*, *ant*(*2″*)*-Ia*, *ant*(*3″*)*-IIc*	*adeABC*, *adeIJK*, *adeFGH*, *abeM*, *amvA*, *abeS*, *abaF*, *abaQ*, *floR*, *macAB*	S83L	S80L
M19	164	164	1418	234	*bla*_OXA-91_	*aph*(*3′*)*-VI*, *bla*_NDM-1_, *bla*_ADC-257_^a^, *bla*_CARB-16_, *ant*(*2″*)*-Ia*, *ant*(*3″*)*-IIc*	*adeABC*, *adeIJK*, *adeFGH*, *abeM*, *amvA*, *abeS*, *abaF*, *abaQ*, *macAB*	S83L	S80L
M20	570	2	1701	546	*bla*_OXA-66_	blaOXA-23, *aph*(*3′*)*-VI*, *bla_NDM-1_*, *bla_ADC-73_*, *aph*(*3′*)*-Ia*, *bla_TEM-1_*, *aph*(*3″*)*-I*, *aac*(*6′*)*-Ib’*, *catB8*, *ant*(*3″*)*-Ia*, *sul1*, *ArmA*, *msr*(*E*), *mph*(*E*), *ant*(*3″*)*-IIc*	*adeABC*, *adeIJK*, *adeFGH*, *abeM*, *amvA*, *abeS*, *abaF*, *abaQ*, *macAB*	S83L	S80L

a Novel ADC allele.

b QRDR, quinolone resistance determining regions.

Analysis of the nucleotide sequence of *pmrABC* and *IpxACD* genes of the colistin-resistant isolate (M19) and comparison to their wild-type alleles in *A. baumannii* ATCC 19606 revealed the existence of multiple mutations. These included point mutations in the histidine kinase gene *pmrB* (H89L) and mutations in *pmrC* (I42V, I212V, R323K, A354S, and V470I). Only silent mutations were identified in *pmrA*. Within *IpxACD* genes, point mutations were identified in *IpxA* (Y131H and Y231H), *IpxC* (C120R, N287D, and K130T), and *lpxD* (V631 and E117K). Further analysis of genomic mutations revealed that levofloxacin resistance in all isolates was promoted by amino acid substitutions in quinolone resistance determining regions (QRDRs) of both DNA gyrase (S83L) and topoisomerase (S80L) enzymes.

Multidrug efflux pumps, including members of the major facilitator superfamily (MFS) and resistance-nodulation-division (RND) family and additional multidrug efflux pumps, were identified in the isolates. Susceptibility profiles of the *bla*_NDM_-positive isolates and resistance determinants carried by each are shown in Figure 2.

### Insertion Sequences

**Figure 3:**
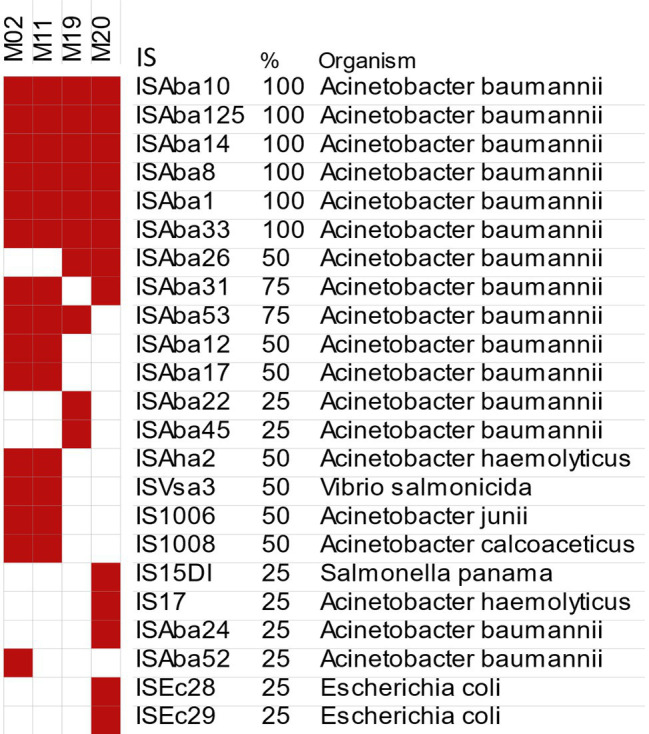
Genome-wide distribution of different IS elements in the *bla*_NDM_-positive isolates predicted by ISfinder. Red and white colors denote the presence and absence of each IS element, respectively.

Investigating the insertion sequences using ISfinder revealed the existence of at least 24 IS elements distributed throughout the genomes. Most of them originated from *A. baumannii* and other *Acinetobacter* species. Only four IS elements were acquired from other bacterial species, such as *Escherichia coli*, *Vibrio salmonicida*, and *Salmonella panama*. Six types of ISs were conserved in all isolates, including IS*Aba*1, IS*Aba*8, IS*Aba*10, IS*Aba*14, IS*Aba*33, and IS*Aba*125. The diversity of IS content of the four genomes and their microbial origins are depicted in Figure 3.

### Genetic Context of Resistance Genes

**Figure 4:**
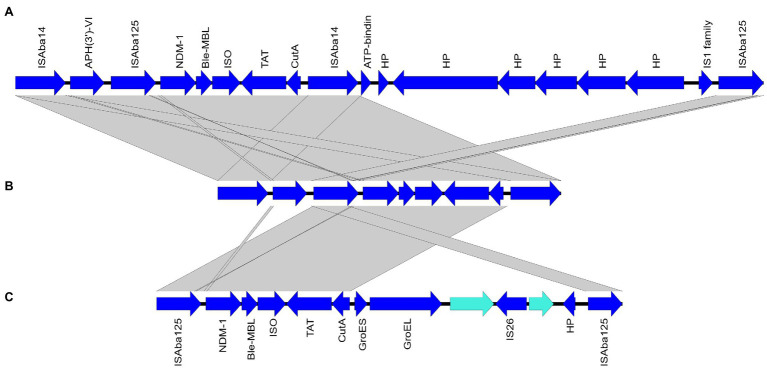
Graphical representation of *bla*_NDM-1_ genetic environment in isolates M02 and M11 **(B)** compared to the closest match sequence *Tn*125-like transposon of *A. baumannii* strain ACN21 (GenBank accession: CP038644.1) **(A)** and *Tn*125 (GenBank accession: KF702386.1) **(C)**. ORFs orientation is indicated by arrows. Grey bands between panels indicate more than 98% sequence similarity. Genes are labelled by their protein products; NDM-1, New Delhi metal-beta-lactamase enzyme; ble-MBL, bleomycin resistance protein; ISO, phosphoribosylanthranilate isomerase; TAT, twin-arginine translocation pathway signal sequence protein; CutA, divalent cation tolerance protein; HP, hypothetical protein; GroES, co-chaperonin protein; GroEL, type I chaperonin.

Whole-genome sequencing results revealed that *bla*_NDM-1_ genes were carried on the chromosomes of all sequenced isolates. Analysis of the immediate genetic environment of the *bla*_NDM-1_ gene revealed the existence of IS*Aba14* upstream to the divalent cation tolerance protein (CutA)-coding gene in the isolates M2, M11, and M20 in addition to the IS*Aba125* element upstream to *bla*_NDM-1_. This genetic organization is similar to that of Tn*125*-like transposon previously reported by Bonnin et al. (2013). BLAST analysis of the contigs harboring *bla*_NDM-1_ showed highest similarity to the chromosome of *A. baumannii* strain ACN21 (GenBank accession: CP038644.1; Vijayakumar et al., 2020). Using this genome as a reference for Islandviewer analysis showed an upstream amikacin resistance gene (*AphA6*) and another copy of IS*Aba14* in ST85^Pas^ isolates (M02 and M11). This was further confirmed by mapping raw sequencing reads of such isolates against a larger segment of *A. baumannii* strain ACN21 chromosome. This genetic organization was shown in Figure 4 together with a comparative genetic analysis of Tn*125*-like transposon and Tn*125* (GenBank accession: KF702386.1). Similar analysis failed to localize *AphA6*-IS*Aba14* in the upstream region of the *bla*_NDM-1_-harboring transposon in M20. Different genetic environment was noted for *bla*_NDM-1_ carried by M19 in which the upstream IS*Aba125* element was immediately preceded by IS1 family transposase in an organization with no similarity in the NCBI nucleotide database. Furthermore, interruption of the right hand of the transposon by IS*Aba14* could not be concluded.

**Figure 5:**
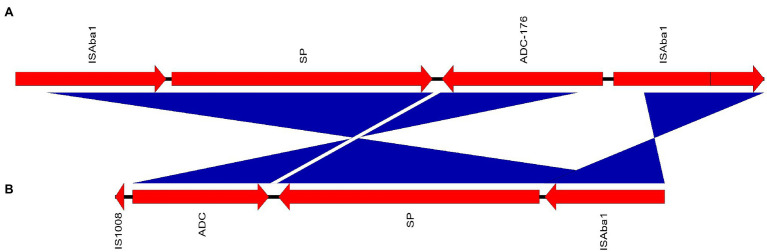
Gene maps showing the genetic environment of *IS1008*-interrupted *bla*_ADC_ carried by M11 **(A)** compared to *A. baumannii* ACN21 chromosome (GenBank accession: CP038644.1) **(B)**. ORFs orientation is indicated by arrows. Blue bands between panels indicate inverted sequences with more than 98% sequence similarity. Genes are labelled by their protein products; ADC, *bla*_ADC_ gene disrupted by IS6; SP, signal peptide.

Analysis of the intrinsic *bla*_ADC_ genes and their association with upstream insertion elements revealed that the novel allele *bla*_ADC-257_ carried by M19 was preceded by IS*Aba1*. In the isolates M02 and M11, *bla*_ADC_ genes were interrupted by IS1008 family transposase leading to missing N-terminus. Hence, the *Acinetobacter*-derived cephalosporinase variant could not be identified. The IS1008-interrupted gene had no similarity in the NCBI nucleotide database. The context of the interrupted gene compared to the closest match sequence (*A. baumannii* strain ACN21 chromosome) is shown in Figure 5. Similarly, *bla*_ADC-73_ with missing N-terminus was harbored by M20, while the disrupting sequence could not be identified.

*bla*_OXA-23_ carried by M20 was found to be embedded within Tn*2006* in which it was bracketed by IS*Aba1*, while *bla*_OXA-94_ in M02 and M11 was preceded by IS*Aba1* element in a reverse orientation. On the other hand, *bla*_OXA-91_ and *bla*_OXA-66_ carried by the isolates M19 and M20 had no upstream insertion sequences.

Using *A. baumannii strain* MS14413 chromosome (GenBank: CP054302.1) as a reference for Islandviewer analysis, a 20,844bp genomic resistance island that showed 99.62% identity to *A. baumannii* genomic resistance island 3 (AbGRI3, accession number: KX011025.2) was identified in M20. The resistance island hosted the resistance genes: *aph*(*3`*)*-Ia*, *aac*(*6`*)*-Ib`*, *catB8*, ant(3*``*)*-Ia*, *sul1*, *ArmA*, *msr*(*E*), and *mph*(*E*) bracketed by IS26 family transposases.

In all isolates carrying *ant*(*2``*)*-Ia* (*aadB*), the gene was found on pRAY plasmid (6,076bp) derivatives. A plasmid sequence identical to pRAY*-v1 (GenBank accession: JF343536) was identified in M19, while those carried by M02 and M11 showed 100% identity to pRay* (GenBank accession: JQ904627). No other resistance plasmids were identified in our isolates.

**Figure 6:**
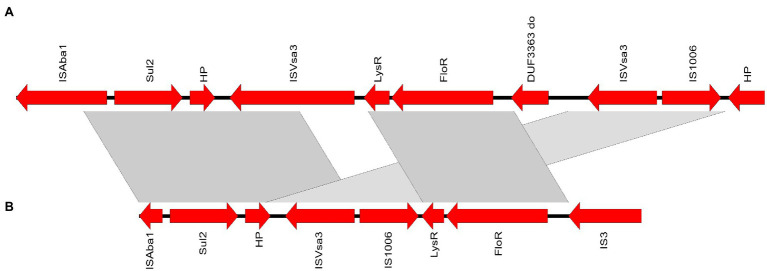
Depiction of the genetic structure containing the genes *sul2* and *floR* identified in strain M11 **(A)** compared to *Acinetobacter indicus* strain GXNN62X4 chromosome, GenBank accession: CP071319 **(B)**. ORFs orientation is indicated by arrows. Grey bands between panels indicate more than 98% sequence similarity. Genes are labelled by their protein products; LysR, LysR family transcriptional regulator.

The chloramphenicol resistance gene, *floR* harbored by the isolates M02 and M11, was linked to a genetic structure containing *sul2*. Both were flanked by insertion elements with the order IS4, *Sul2*, hypothetical protein-coding gene, IS*Vsa*3, IS1006, *LysR*, *floR*, and IS3. The closest match to this region was shown by *Acinetobacter indicus* chromosome (GenBank accession: CP071319.1). The genetic structure containing *floR* and *sul2* genes compared to the closest match sequence is depicted in Figure 6.

Macrolide resistance genes *msr*(*E*) and *mph*(*E*) were flanked by an upstream ISNCY family transposase and a downstream IS*Aba1* element. A genetic organization that is identical to that carried by *A. baumannii* strain ACN21 chromosome (GenBank accession: CP038644.1).

## Discussion

A threatening rise in the incidence of carbapenem-resistant *A. baumannii* has been increasingly reported worldwide (Levy-Blitchtein et al., 2018; Moghnieh et al., 2018; Alcantar-Curiel et al., 2019) and in Egypt as well (Al-Hassan et al., 2019; Benmahmod et al., 2019; Mabrouk et al., 2020), leaving behind a substantial number of difficult to treat infections. For a deeper insight into the molecular mechanisms underlying carbapenem resistance in this highly problematic pathogen, a collection of 20 *A. baumannii* clinical isolates was screened for carbapenemase-coding genes by PCR. Four NDM producers were identified in clinical specimens recovered from ICU patients with severe infections. XDR phenotype was identified in all *bla*_NDM_-positive *A. baumannii* with few reserved therapeutic options. These included tigecycline, colistin (for M02, M11, and M20), and amikacin (for M19) frequently associated with unfavorable pharmacokinetics and/or adverse effects particularly in critically ill patients (Spapen et al., 2011).

Draft genomes of the *bla*_NDM_-positive isolates were obtained by Illumina sequencing for subsequent MLST and resistome analysis. *In silico* MLST and goeBURST analysis revealed that three out of four NDM producer *A. baumannii* belonged to the high-risk international clones (ICs), known for outbreak potential, worldwide dissemination (Karah et al., 2012), and multidrug resistance (Diancourt et al., 2010). M02 and M11 were assigned ST85^Pas^/1089^Oxf^ that belong to IC9, recently described by Müller et al. (2019). Abundance of studies reporting *bla*_NDM-1_-positive *A. baumannii* of ST85^Pas^ from Middle East countries (Bonnin et al., 2013; Decousser et al., 2013; Rafei et al., 2014; Salloum et al., 2018) has drawn attention on its probable endemicity in this region. IC2 was represented only by M20 (ST570^Pas^/1701^Oxf^), whose genome was loaded by the highest share of resistance genes. The abundance of IC2 *A. baumannii* in Egypt was also reported by others (Al-Hassan et al., 2019; Wasfi et al., 2021). To the best of our knowledge, this is the first report of *bla*_NDM_-positive *A. baumannii* strain that belongs to ST164^Pas^/ST1418^Oxf^. Although MDR-resistant *A. baumannii* isolates that belong to ST164^Pas^ have been increasingly reported from different parts of the world (Coelho-Souza et al., 2013; Loraine et al., 2020; Tada et al., 2020), none was reported to carry a *bla*_NDM_ gene.

The SNP-based phylogeny analysis (Figure 1) showed that the isolates M02 and M11 were genetically related to two *bla*_NDM_-positive *A. baumannii* strains of the same sequence type (1089^Oxf^/85^Pas^). These included *A. baumannii* strain Cl300 isolated in Lebanon in 2015 and strain ACN21 isolated in India in 2018. M20 was found to be genetically related to two NDM producer *A. baumannii* strains isolated in United States in 2016 (TP2 and TP3). Both TP2 and TP3 had the Oxford ST1578 a double locus variant of ST1701 to which M20 belongs. On the other hand, M19 showed no genetic relatedness to any of the NDM producer *A. baumannii* strains for which complete genomes were available in the NCBI. Inclusion of four draft genomes that belong to ST164^Pas^ and ST1418^Oxf^ retrieved from PubMLST genome collection revealed that M19 was most genetically related to *A. baumannii* strain CPO20190206 isolated from Denmark (ST164^Pas^) and *A. baumannii* strain JorAb40 isolated from Jordan (ST1418^Oxf^). Both strains were isolated in 2019 and, interestingly, none was found to carry a *bla*_NDM_ gene.

Resistome analysis disclosed a wide arsenal of resistance genes presented in Table 2 and correlated with the susceptibility profiles in Figure 2. Both intrinsic and acquired resistance mechanisms contributed to β-lactams resistance. The carbapenem-hydrolyzing class D β-lactamases (oxacillinases) provide both intrinsic (*bla*_OXA51-like_ genes) and acquired (*bla*_OXA-23, 40, 58, 143,235_-like genes) resistance to β-lactams including carabapenems (Poirel and Nordmann, 2006; Ghaith et al., 2017). Overexpression of OXA-type β-lactamases has been linked to an upstream IS element, most frequently IS*Aba1*, through which an additional promotor is provided (Evans and Amyes, 2014). *bla*_OXA-94_ preceded by IS*Aba1* element was identified in M02 and M11 while no IS elements could be identified upstream to *bla*_OXA-91_ or *bla*_OXA-66_ carried by M19 and M20, respectively.

In addition to intrinsic OXA-type β-lactamases, the IC2 isolate (M20) also carried *bla*_OXA-23_ the most widely disseminated oxacillinase acquired by carbapenem-resistant *A. baumannii* (Mugnier et al., 2010). Association of *bla*_OXA-23_ with IC2 *A. baumannii* has been reported worldwide (Hamidian and Nigro, 2019). As with other IC2 isolates, *bla*_OXA-23_ carried by M20 was found to reside in Tn*2006* in which the gene is bracketed by two inversely oriented IS*Aba1* elements. Tn*2006* is the most common structure harboring *bla*_OXA-23_ either alone or incorporated into AbGRIs (Hamidian and Nigro, 2019).

Although the association of *bla*_OXA-91_ and *bla*_NDM-1_ in *A. baumannii* was not previously described in Egypt, co-existence of *bla*_OXA-51_-like, *bla*_OXA-23_ and *bla*_NDM-1_ was reported by Wasfi et al. (2021).

Analysis of the genetic environment of *bla*_NDM-1_ in the sequenced isolates showed different environments in different sequence types. IS*Aba14* element was inserted upstream to the *cutA* gene in M02, M11, and M20. This was previously documented by Bonnin et al. (2013) who failed to identify a downstream second copy of IS*Aba125* by PCR and suggested loss of functionality of this truncated transposon (ΔTn*125*). WGS of *bla*_NDM_-positive isolates by a later study (Vijayakumar et al., 2020) uncovered the existence of a second copy of IS*Aba125* downstream to the IS*Aba14*-interrupted transposon. Interestingly, analysis of the upstream region to the truncated transposon revealed the existence of the amikacin resistance gene *AphA6* preceded by another copy of IS*Aba14* in ST85^Pas^ isolates (Figure 4). The two IS*Aba14* elements were thus thought to form an alternative composite transposon in which two resistance genes were enclosed for transposition (*bla*_NDM-1_ and *AphA6*) rather than the widely known Tn*125* in which *bla*_NDM-1_ was hosted as the sole antimicrobial resistance gene. Transposition of this composite transposon might, therefore, favor the co-transfer of resistance to two of the last-line antimicrobial treatment options for MDR and XDR *A. baumannii*. Nevertheless, experimental analysis is required to examine the transposition potential of this transposon. In M19, IS1 family transposase was identified immediately upstream to IS*Aba125* that precedes the *bla*_NDM-1_ gene. Insertion of IS1 element in this location was not identified in the nucleotide database of the NCBI.

Intrinsic to all *A. baumannii*, cephalosporin resistance is mediated by ADC (formerly known as *bla*_AmpC_). In addition to the incomplete *bla*_ADC-73_ carried by M20, a novel *bla*_ADC_ allele (*bla*_ADC-257_) with an upstream *ISAba1* element was identified in M19 recovered from a blood culture of a female patient admitted to the ICU with fever of unknown origin. With no similarity in the NCBI nucleotide database, *bla*_ADC_ genes carried by M02 and M11 were interrupted by an IS1008 element (Figure 5). No alternative intact copies of *bla*_ADC_ were identified in M02, M11, or M20. Other β-lactamases identified here included class A β-lactamases, more efficiently capable of hydrolyzing penicillins and cephalosporins than carbapenems (Jeon et al., 2015). These were coded by *bla*_Tem-1_ carried by M20 and *bla*_CARB-16_ in M19. However, their association with mobile elements could not be clearly determined.

In addition to the intrinsic aminoglycoside resistance gene *ant*(*3``*)*-IIc* (Zhang et al., 2017), the amikacin-modifying enzyme-coding gene *aph*(*3*`)*-VIa* (*aphA6*) was found in all isolates. The predominance of *aph*(*3*`)*-VIa* among the aminoglycoside modifying enzymes-coding genes was also reported by others (Aghazadeh et al., 2013; Sheikhalizadeh et al., 2017). Notably, the gene was also identified in the amikacin-sensitive isolate M19. Identification of *aph*(*3*`)*-VIa* in amikacin-susceptible isolates was also reported before (Aghazadeh et al., 2013; Sheikhalizadeh et al., 2017). In *ant*(*2``*)*-Ia*-positive isolates, the gene was found in pRAY plasmid variants. pRAY is a 6 Kb plasmid widely distributed in *Acinetobacter* species comprising the most common resistance mechanism to gentamicin and tobramycin (Hamidian et al., 2012).

Acquired 16S rRNA methyltransferases constitute the most important aminoglycoside resistance mechanism conferring resistance to most of the clinically important aminoglycosides (Galimand et al., 2003). Of them, *armA* has been widely reported from *A. baumannii* particularly those of the IC2 (Blackwell et al., 2017). Within a 20,844bp genomic resistance island closely similar to *A. baumannii* genomic resistance island 3 (AbGRI3; Blackwell et al., 2017), *armA* gene was identified in M20 (IC2). Other resistance genes hosted by the genomic island include *aph*(*3`*)*-Ia*, *aac*(*6`*)*-Ib`*, *catB8*, ant(3*``*)*-Ia*, *sul1*, *msr*(*E*), and *mph*(*E*). Another unique genetic structure in which genes coding resistance to two different antimicrobial classes was identified in M02 and M11 (Figure 6). This included the chloramphenicol efflux pump (FloR)-coding gene and *sul2*, conferring resistance to sulfamethoxazole/trimethoprim, enclosed by insertion elements. The closest match to this region was shown by *Acinetobacter indicus* chromosome (GenBank accession: CP071319.1) from which it may have been acquired with some genetic rearrangement.

In the absence of plasmid-mediated quinolones resistance genes, nonsusceptibility to levofloxacin in all NDM producers investigated here was mediated by target site mutations. These affected the QRDRs within GyrA (S83L) and ParC (S80L) enzymes. The mutation pattern identified in our isolates was commonly reported as the predominant mechanism responsible for fluoroquinolones resistance in *A. baumannii* (Hamed et al., 2018; Nodari et al., 2020; Roy et al., 2021).

Resistance to colistin, the last line of defense against XDR *A. baumannii*, was evident in only one isolate (M19) that, fortunately, retained susceptibility to amikacin and tigecycline. Colistin resistance in M19 was accompanied by multiple nonsynonymous mutations affecting *pmrABC* and *IpxACD* genes. Missense mutations identified in *pmrB* (H89L) and *pmrC* (I42V) genes carried by M19 were also reported in colistin-resistant *A. baumannii* studied by Nurtop et al. (2019) in Turkey. It is worth mentioning that the amino acid affected by *pmrB* mutation identified here is located outside the histidine kinase domain, the main determinant of colistin resistance in *A. baumannii* (Arroyo et al., 2011; Beceiro et al., 2011; Lesho et al., 2013). Moreover, all *lpxACD* mutations identified here were previously reported in both colistin-susceptible and colistin-resistant isolates (Oikonomou et al., 2015; Haeili et al., 2018; Nurtop et al., 2019). Accordingly, novel unidentified resistance mechanisms might stand behind the high-level resistance (MIC≥128μg/ml) of M19 to colistin. Further investigations including gene expression analysis are therefore required to confirm or role out the impact of such mutations on colistin susceptibility.

Diverse efflux pumps, whose overexpression has been linked to multidrug resistance, were identified in the sequenced isolates. RND efflux pumps known by their broad substrate profiles (Coyne et al., 2011), including AdeABC, AdeIJK, and AdeFGH. were identified in all isolates. RND efflux pumps contribute to intrinsic resistance of *A. baumannii* to several classes of antimicrobials. Other multidrug efflux pumps carried by all isolates included AbeM, a member of the multidrug and toxic compound extrusion family efflux pumps and the small multidrug resistance efflux pump AbeS (Coyne et al., 2011). Except for FloR conferring resistance to phenicols in M02 and M11 only, efflux pumps of the MFS were disseminated in all sequenced genomes. With narrow substrate profiles, AmvA, AbaF, and AbaQ are known to extrude erythromycin, fosfomycin, and quinolones, respectively (Coyne et al., 2011; Perez-Varela et al., 2018). The macrolide-specific ABC pump MacAB was also found in all isolates.

It is worth mentioning that the current study suffers from some limitations, most importantly is using short-read sequencing technology instead of a hybrid long- and short-read sequencing approach known to produce more accurate genome organization. Consistent with other studies (Leal et al., 2020), resistance to some antimicrobials could not be correlated to known resistance genes highlighting the need for further investigations including gene expression analysis or identification of novel resistance determinants. Finally, only four genomes were sequenced here thus correlating resistance genes with particular STs could not be fully achieved.

## Conclusion

The current study is one of the few studies reporting WGS of *A. baumannii* clinical isolates from Egypt. The isolates showed XDR phenotype and were recovered from ICU patients. High-risk international clones were identified, predominantly IC9 (ST85^Pas^) widely reported from Middle East countries. Diverse mobile elements were associated with resistance genes with novel insertion sites and genetic organizations. Co-existence of amikacin and carbapenem resistance genes on an *ISAba14*-bracketed transposon was uniquely identified in ST85^Pas^/ST1089^Oxf^. *bla_NDM-1_* gene was identified, for the first time, on the chromosome of an *A. baumannii* strain that belongs to sequence type ST164^Pas^/S1418^Oxf^. WGS of the highly problematic MDR and XDR pathogens may aid in the identification of emerging resistance genes and their dissemination dynamics. Co-existence of resistance genes within mobile genetic elements could also be identified. This may aid in optimizing treatment guidelines to avoid selection of resistance to last-line antimicrobials. WGS also permits monitoring the emergence of novel global MDR clones and facilitates comparative genomic analysis and developing cheaper molecular techniques for routine screening.

## Ethical Approval

The study was performed in accordance with relevant guidelines and regulations, and no experiments were performed on humans and/or human tissue samples. The study was approved by the local Ethical Committee of clinical and chemical pathology department, Kasr Al-Aini Hospital, Cairo university. Only bacterial isolates were collected for the routine laboratory work to ensure patient care and informed consents were not required.

## Data Availability Statement

The datasets presented in this study can be found in online repositories. The names of the repository/repositories and accession number(s) can be found in the article/Supplementary Material.

## Author Contributions

MZ, AH, MA, HR, and SH contributed to the study design, performance of experiments, and data analysis. SH performed the genomes assembly and bioinformatic analysis. MZ wrote the first draft of the manuscript. All authors read and approved the final version of manuscript.

## Funding

The authors thank the Deanship of Scientific Research at King Saud University for funding this work through Project No. RGP-038.

## Conflict of Interest

The authors declare that the research was conducted in the absence of any commercial or financial relationships that could be construed as a potential conflict of interest.

## Publisher’s Note

All claims expressed in this article are solely those of the authors and do not necessarily represent those of their affiliated organizations, or those of the publisher, the editors and the reviewers. Any product that may be evaluated in this article, or claim that may be made by its manufacturer, is not guaranteed or endorsed by the publisher.

## Footnotes

^1^ https://pubmlst.org/organisms/acinetobacter-baumannii/

^2^ https://pubmlst.org/organisms/acinetobacter-baumannii/

^3^ http://www.genomicepidemiology.org/

^4^ https://card.mcmaster.ca/analyze/rgi

^5^ http://www.pathogenomics.sfu.ca/islandviewer/

^6^ http://www-is.biotoul.fr

^7^ https://www.ncbi.nlm.nih.gov/sra/

^8^ https://submit.ncbi.nlm.nih.gov/subs/genome/
