# Supplementary material for: Genomic Characterization of Extensively Drug-Resistant NDM-Producing Acinetobacter baumannii Clinical Isolates With the Emergence of Novel blaADC-257
Source: Front Microbiol. 2021 Nov 22;12:736982. doi: 10.3389/fmicb.2021.736982 (PMC8645854; doi:10.3389/fmicb.2021.736982)
Supplement: Supplementary file 1 [file Data_Sheet_1.docx]

Supplementary Material

Supplementary Table 1. Accession numbers of raw reads and draft genomes of the sequenced isolates

| Isolate number | SRA accession number | Genome accession numbers |
| --- | --- | --- |
| M02 | SRX9845557 | JAESHR000000000 |
| M11 | SRX10472612 | JAESHK000000000 |
| M19 | SRX10487148 | JAESHC000000000 |
| M20 | SRX10487179 | JAESHB000000000 |

SRA, sequence read archive (<https://www.ncbi.nlm.nih.gov/sra/>).

Supplementary Table 2. Post-assembly and annotation metrics of all sequenced genomes

| Isolate No. | Post-assembly Metrics | | | | | Annotation Metrics | | | | |
| --- | --- | --- | --- | --- | --- | --- | --- | --- | --- | --- |
|  | No. of contigs | Total length | GC (%) | N50 | Genome coverage | Total genes | Total CDSs | Coding genes | CDSs with proteins | RNA genes |
| M02 | 455 | 3919334 | 39.19 | 16577 | 16X | 4,068 | 3,996 | 3,878 | 3,878 | 72 |
| M11 | 690 | 3834431 | 39.49 | 10028 | 24X | 3,823 | 3,761 | 3,628 | 3,628 | 62 |
| M19 | 908 | 3773846 | 39.55 | 7080 | 42X | 3,870 | 3,808 | 3,642 | 3,642 | 62 |
| M20 | 481 | 3899209 | 39.41 | 15553 | 42X | 3,873 | 3,796 | 3,700 | 3,700 | 77 |

**
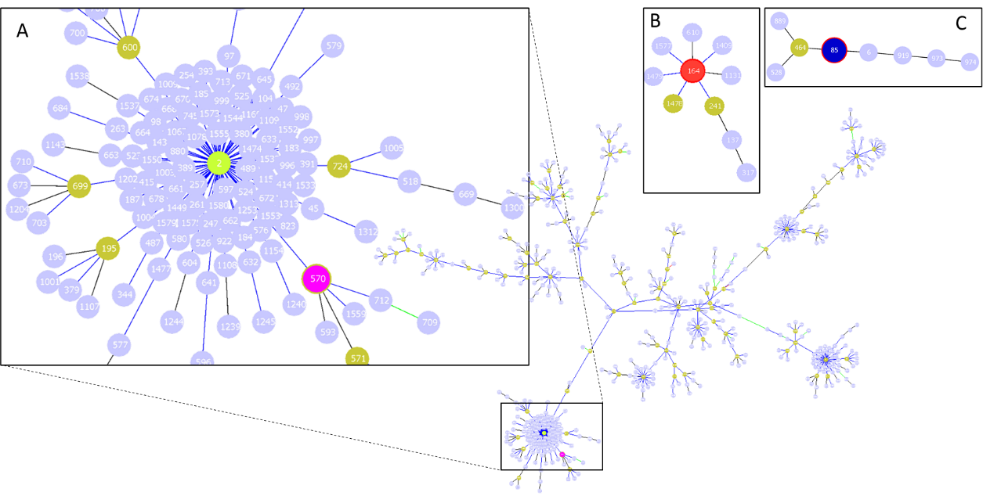
**

Supplementary Figure 1. Minimum Spanning Tree diagram of *bla*_NDM_-positive isolates STs together with other STs in MLST database (Pasteur scheme). STs are indicated by the numbers inside each circle. Purple, red and blue circles correspond to the STs to which the *bla*_NDM_--positive isolates belong. ST570^Pas^ identified in isolate M20 belongs to CC2 (A). Isolate M19 had the ST/CC164^Pas^ (B) while ST85^Pas^ (Isolates M02 an M11) was found to belong to CC464 (C).
